# Towards a unified data infrastructure to support European and global microbiome research: a call to action

**DOI:** 10.1111/1462-2920.15323

**Published:** 2020-12-03

**Authors:** Matthew J. Ryan, Michael Schloter, Gabriele Berg, Linda L. Kinkel, Kellye Eversole, James A. Macklin, Daria Rybakova, Angela Sessitsch

**Affiliations:** ^1^ Genetic Resources Collection CABI Egham UK; ^2^ Helmholtz Zentrum München National Research Center for Environmental Health, Research Unit for Comparative Microbiome Analysis Oberschleissheim Germany; ^3^ Institute of Environmental Biotechnology Graz University of Technology Graz Austria; ^4^ Department of Plant Pathology University of Minnesota Saint Paul MN USA; ^5^ International Alliance for Phytobiomes Research Lee's Summit MO USA; ^6^ Eversole Associates Bethesda MD USA; ^7^ Ottawa Research and Development Centre Agriculture and Agri‐Food Canada Ottawa ON Canada; ^8^ AIT Austrian Institute of Technology Center for Health and Bioresources, Bioresources Unit Tulln Austria

## Abstract

High‐quality microbiome research relies on the integrity, management and quality of supporting data. Currently biobanks and culture collections have different formats and approaches to data management. This necessitates a standard data format to underpin research, particularly in line with the FAIR data standards of findability, accessibility, interoperability and reusability. We address the importance of a unified, coordinated approach that ensures compatibility of data between that needed by biobanks and culture collections, but also to ensure linkage between bioinformatic databases and the wider research community.

## Introduction

Microbiology research relies upon access to high‐quality data and associated metadata on microorganisms. Importantly, this includes provenance information concerning the details of the isolation and management of the organism and the link to any data generated from phenotypic or genomic tests. Approaches to data management in biobanks, museums and culture collections often differ, and this reflects the various mechanisms by which these institutions operate and samples are stored, processed and distributed. The rapid development of the microbiome research field has led to additional requirements as the need to manage microbiome resources is different to those required for axenically cultured microorganisms, human material or museum specimens. The microbiome encompasses all of the microbial components in a given ecosystem or plant, animal or human system and has been recently defined by Berg *et al*. (2020)). Ryan *et al*. (2020) addressed the biobanking infrastructure requirements and identified further developmental needs in order to make these suitable for microbiome biobanking. A similar challenge and need exists for the management of microbiome data, as biobank data tend to revolve around single species rather than the datasets associated with communities of organisms. In this paper, we review the current status quo of data management and look at the specific requirements for microbiome research and how this may be achieved, and how the gap between biobank and bioinformatic resources may be bridged.

## The status quo

Culture collections and biobanks have different approaches to the management of data associated with specimens. For biobanks, the International Society for Biological and Environmental Repositories (ISBER; https://www.isber.org/) developed the Standard PREanalytical Code (SPREC) (Benson *et al*., 2011) which ‘identifies and records the main pre‐analytical factors that may have impact on the integrity of sampled clinical fluids and solid biospecimens and their simple derivatives during collection, processing and storage’ (Lehmann *et al*., 2012). In culture collections, data about the cultured organism are typically held as a microbial data set (Table 1). Without such information, samples in a collection are effectively worthless. For current culture collection holdings, the global catalogue of microorganisms (GCM; http://gcm.wfcc.info/) provides a good example of a comprehensive database and information retrieval, analysis and visualization system for microbial resources established through the world data centre for microorganisms (Wu *et al*., 2013). However, the GCM brings together the holdings of culture collections of axenic microorganisms and will need to extend their data model beyond a single organism cultured from a sample to cover a complexity of microbiome subsampling relationships while maintaining links to associated genomic data sets.

**Table 1:** Example of a typical culture collection minimal microbial data set.

Field	Description	Example
Culture collection number	Unique identifier	*IMI 123456*
Organism name	Current accepted taxonomic name	*Aspergillus brasiliensis*
Other or previous accepted name	Any previous taxonomic name associated with the organism (e.g. name changes)	*Aspergillus niger*
Date collected	Date sample collected from the environment	01/05/70
Date isolated	Date strain isolated	01/06/70
Geographical location	Location, coordinates of sample	USA
Isolated from	Host or substrate	Blueberry
Habitat associated with environment	For example desert, arable farm, etc	Farm
Isolated by	Name of person who performed the isolation	A.N. other
Deposited by	Name of person who made the deposition	A.N. other
Other collections	Other institution's strain number where a replicate culture is held	For example NBRC1234
Preservation history	Details of preservation method and associated dates	Cryopreservation, oil
Biosecurity	Organism‐specific biosecurity risk	Low risk
Biosafety	Organism‐specific biosafety risk	Biosafety level 1
Security	Restrictions of the culture collection for release, for example Nagoya Protocol	None
Molecular tests	Sequences	Nucleotide (GenBank): FJ195348 ITS including 5.8S rRNA gene
Proteomic tests	Protein sequences and spectra	None

## Requirements for microbiome

Although there are similarities for ‘axenically’ held strains (e.g. the importance of collection data), a complete reassessment of requirements to meet the needs of the microbiome research community is required and this will require new data fields beyond those currently used in culture collections (Table 2). It is likely that preservation and storage approaches and regimes may be different (Ryan *et al*., 2020), requiring modification of protocols and procedures. New fields may also need to encompass the additional ethical and regulatory requirements, which are necessary depending on the source of the sample and the objectives of the microbiome research. Of key importance will be the use of unique identifiers on microbiome subsampling events, isolated organisms and sequence data to support linking and data provenance. As well as the ongoing need for the standardization of metadata and infrastructure associated with cultures of axenic microbes in culture collections and biobank tissue specimens, there is a need for (standardized) metadata and infrastructure associated with microbiomes and the two need to be aligned. A key question to address is metadata infrastructure needed only for microbiomes for which there are samples that are deposited for future study or reference, or for all microbiome data that are rapidly being generated globally, not just those stored for future use? A few global groups who have microbiome samples deposited and metadata platforms already in place have endeavoured to address some issues but they are distinct from biobanks and culture collection approaches. For example, the U.S. National Ecological Observatories Network (NEON; https://www.neonscience.org/) has a microbiome archive with associated metadata (Yilmaz *et al*., 2011).

**Table 2:** Additional fields that may be required for a minimal microbiome data set^a^.

Quantity of material deposited	Volume/weight of material (as resource may be finite)
Number of replicates available	The number of environmental replicates preserved/available
Reason for deposit	Medical, conservation, research integrity/publication, legal
Type (format) of material deposited	For example rhizoplane, soil, skin, seed, gut, etc.
Metagenomic DNA	DNA deposit available?
Precise location of collection	Importance as microbiomes can vary on an individual or between plants in the same field/location
Supply conditions	Criteria under which subsamples/archival material (which may be finite) are provided to researchers

^a^ Although these fields are common to many environmental datasets, they are over and above the requirements traditionally used in culture collections.

Similar to other genomic data, there is a need to store the raw sequence data and associated metadata from microbiome samples to preserve it for future analysis and reuse. This along with provenance information are critical underpinning components of reproducible science. For information associated with sequences, the International Genomic Standards Consortium established MiXS standards for metadata for microbes (including microbial isolates and covering several microbiome systems) (Yilmaz *et al*., 2011). This standard requires core information on the sampling event including geolocation, the sequencing methodology as well as fields specific to data type and a range of optional environmental packages to capture core measurements defining a broad range of habitats: water, soil, host‐associated, etc. (Field *et al*., 2011).

The metadata associated with the enormous amount of genomic and proteomic data originating from microbiome samples is an important consideration. Often, a small subset of this data is deposited in global sequence data information repositories, without link to the original voucher specimen, material or DNA/RNA sample as the fields containing this information are not mandatory. Once the link between data and sample is broken, work cannot be repeated or reproduced, which compromise the stringency and integrity of the data. There are international attempts to underpin microbiome data storage and interoperability, for example, the mission of the Integrated Microbial Genomes and Microbiomes (IMG/M) system is to support the annotation, analysis and distribution of microbial genome and microbiome datasets sequenced at DOE's Joint Genome Institute (JGI) (Chen *et al*., 2019). Similarly, the U.S. National Microbiome Data Collaborative, which has developed connections to EU efforts in microbiome science and has acquired significant funding to develop a microbiome database capacity, is working to develop a larger international framework and partnership. Dundore‐Arias *et al*. (2020) have addressed the needs for community‐driven metadata standards for agricultural microbiome research to ensure that metadata is consistent and well‐annotated.

Metagenomic libraries may also serve as a repository of functional microbiomes and assembled (predicted) genomes from so far uncultured microorganisms. It has been emphasized that open access metagenomic libraries should be an openly available reference source of microbiomes similar to microbial strain collections (Neufeld *et al*., 2011). The first of such kind was established in the Canadian MetaMicroBiome Library (CM2BL; http://cm2bl.org). The CM2BL is a publicly accessible collection of metagenomic libraries and represents microbiomes of terrestrial and aquatic environments. The sequence database information of these libraries facilitated the researchers to choose relevant libraries for research projects.

## Summary recommendations and the way forward

This is a call for a coordinated, community action. There is a need to build on the best practice used by both biobanks and culture collections (and also environmental repositories) in association with the European Bioinformatics institute, Elixir (an EU infrastructure whose goal is to coordinate resources so that they form a single infrastructure), the International Genomic Standards Consortium and similar initiatives, while recognizing the need to ensure metadata is compatible for bioinformatic uses. While no common standards exist, reference to the FAIR data standards (Wilkinson *et al*., 2016) of findability, accessibility, interoperability and reusability (endorsed by the G20 nations) will be a good starting point. The intention should be to make all data open access along the Global Open Data for Agriculture and Nutrition (GODAN) model (Musker *et al*., 2018), but barriers must be overcome. For example, issues related to IP protection, the fair and equitable sharing of benefits under the Nagoya Protocol of the Convention of Biodiversity and, industry often wanting free access to databases while often restricting access to their own commercially valuable data.

While both culture collections and biobanks have remits and responsibility to meet the needs of the microbiome research community, the current data infrastructure is extremely limited and fragmented and not coordinated to support microbiome research. Therefore, there is an urgent requirement to assess the strategic benefits of coordinating and establishing a common data infrastructure to underpin the quality and reproducibility of all microbiome‐based research for both academic and commercial applications. This should encompass whether biobanks, culture collections or both can be further expanded to cover this area, while also considering current legislation – and adaptations of it, data handling – quality and provenance and quality and standard operating protocols. This will require the identification of infrastructural overlaps in order to gauge what is missing and what is required within the EU and beyond. Key international umbrella organizations such as ISBER, European and African Society for Biobanking, Word Federation for Culture Collections and Global Genome Biodiversity Network (GGBN) (http://www.ggbn.org/ggbn_portal/) will need to be consulted to measure what data are accessible globally and whether these represent ‘total’ or restricted data sets. Consultation with the relevant standards bodies will also be essential in the alignment and inclusion of microbiome data and metadata including the Biodiversity Information Standards (TDWG) maintainers of the GGBN data standard and the Genomic Data Standards Consortium (GSC).

In the future, we will not only have DNA/RNA‐based data but also protein data and metabolomics data. These datasets are stored in different archives with a different history and different requirements for metadata. Thus, sometimes it is not even possible to have the same metadata set for the same sample if genomic, metabolomics or proteomic data are stored. This emphasizes the importance of unique identifiers and the urgent need for unifying principles to allow for easy discovery and interoperability. Information will also likely be not in the same data store, so it is important to keep this data associated to allow interoperability based on standards and infrastructures. Currently, there is little to no linkage so it needs to be facilitated.

Most importantly it is a duty of individual microbiome researchers to actively and accurately record all data produced from their research. Ideally, it should be a condition of publication of their research that standard formats are followed to ensure standardization and reproducibility of their research. Ultimately, there should be a minimal mechanism to address the appropriate data standards required for microbiome research, ensuring compatibility and by bringing the best aspects of the many current data standards and approach together into an open access, universal standardized approach.
